# Supplementary material for: Toxoplasma-proximal and distal control by GBPs in human macrophages
Source: Pathog Dis. 2021 Dec 21;79(9):ftab058. doi: 10.1093/femspd/ftab058 (PMC8752258; doi:10.1093/femspd/ftab058)
Supplement: ftab058_Supplemental_Files [file ftab058_supplemental_files.zip › Fisch_et_al_P&D_SupFigureLegends.docx]

**SUPPLEMETARY FIGURE LEGENDS**

**Figure S1: Growth morphology and differentiation of human macrophage models**

**(A)** Images of human macrophage cell lines before (top) and following indicated differentiation steps (bottom). Human induced pluripotent stem cells (iPSC); embryonic body (EB); peripheral blood monocytic cell (PBMC). Scale bar, 50 μm. Image of whole blood from <https://commons.wikimedia.org/>. **(B)** Flow cytometry measuring CD14, CD16 or CD68 surface expression of the indicated cell line, prior to (black) or post differentiation into macrophages (blue). **(C)** Absolute mRNA copies of indicated *GBP* per cell for IFNγ-primed THP-1, iPSC-derived or monocyte-derived macrophages (MDM) at 16 h post treatment. **(D)** Fold change induction of indicated *GBP* following treatment of indicated cell line with 50 IU mL^-1^ IFNγ for 16 hours, plotted as ∆∆Ct normalizing to untreated cells (ø) and relative to human Hypoxanthine‑guanine phosphoribosyltransferase (*HPRT1*). **(E)** RT-qPCR measurement of *GBP* expression silencing in IFNγ-primed THP-1, iPSCs or MDMs transfected with siRNA against *GBP1-5*. mRNA fold-change normalized to *HPRT1* is indicated as percentage of cells transfected with non-targeting control (CTRL) siRNA. **(F)** Images of HFF plaques formed by Tg obtained following growth in naïve or IFNγ-primed THP-1 cells for 18 hours additionally transfected with siRNAs against the indicated *GBP* or CTRL (top) and quantification of plaque area from images (bottom). Plaque area of Tg normalized to the corresponding naïve condition, to represent the increase of Tg-viability caused by silencing of the respective *GBP*. **Data information:** Graphs in **(B+E)** representative of n = 3 independent experiments or n = 4 different donors (MDMs). Graphs in **(C+D)** show data from n = 14 (THP-1) or n = 10 (iPSC) independent experiments or n = 6 donors (MDMs) and mean ± SEM; n.d. not detected. Images in **(F)** representative of n = 3 independent experiments. ***** P* ≤ 0.0001 in **(F)** comparing to CTRL siRNA transfected cells following adjustment for multiple comparisons.

**Figure S2: Characterization of human GBP CRISPR knockout cell lines**

**(A)** Immunoblots for indicated proteins from IFNγ-primed THP-1 WT, THP-1∆*GBP1*, ∆*GBP2* or ∆*GBP5* cells. **(B)** Fold change of mRNA expression of indicated human *GBP* in IFNγ-primed THP-1 WT, THP-1∆*GBP1*, ∆*GBP2* or ∆*GBP5* cells relative to human Hypoxanthine-guanine phosphoribosyltransferase (*HPRT1*); n.d.: not detected. **(C)** PCR amplification of indicated *GBP* coding sequences (CDS) on cDNA created from mRNA obtained from IFNγ-primed THP-1 WT, THP-1∆*GBP1*, ∆*GBP2* or ∆*GBP5* cells. Yellow arrowhead highlights truncated GBP1 CDS in THP-1∆*GBP1* cells. **(D)** Sanger sequencing of *GBP1* and *GBP5* CDS captured into TOPO-TA vectors following PCR amplification shown in **(C)** aligned to displayed wildtype sequence of respective *GBP* mRNA. **(E)** Overview of sequencing results of human *GBP* CDS from THP-1∆*GBP1*, ∆*GBP2* or ∆*GBP5* cells; n.d. not detected. **(F)** Immunoblots for indicated proteins from IFNγ-primed THP-1 WT, THP-1∆*GBP1*, ∆*GBP2* or ∆*GBP5* cells. **(G)** Immunoblots from THP-1 WT, ∆*GBP1*, ∆*GBP1*+Tet-*GBP1*, ∆*GBP2*, ∆*GBP2*+Tet-*GBP2*, ∆*GBP5* and ∆*GBP5*+Tet-*GBP5* cells treated with IFNγ, and Doxycycline (Dox) as indicated. **Data information**: Images in **(A), (C)** and **(D)** representative of n = 2 experiments. Graphs in **(B)** show mean ± SEM from n = 3 independent experiments. Images in **(G)** representative of n = 3 independent experiments.

**Figure S3: Reconstitution of mutated GBPs into macrophages**

Immunoblots from IFNγ-primed THP-1 WT and ∆*GBP1* **(A)**, ∆*GBP2* **(B)** or ∆*GBP5* cells **(C)** reconstituted with the indicated mutants of GBP1, GBP2 or GBP5 respectively and treated with Doxycycline (Dox) as indicated. *Marks unspecific bands.

**Figure S4: Reconstitution of mutated mCherry-tagged GBPs into macrophages**

Immunoblots from IFNγ-primed THP-1 WT and ∆*GBP1*, ∆*GBP2* or ∆*GBP5* cells reconstituted with the indicated mCherry (mCH)-tagged mutants of GBP1, GBP2 or GBP5 respectively and treated with Doxycycline (Dox) as indicated.
