# Supplementary figures and images for: Toxoplasma-proximal and distal control by GBPs in human macrophages
Source: Pathog Dis. 2021 Dec 21;79(9):ftab058. doi: 10.1093/femspd/ftab058 (PMC8752258; doi:10.1093/femspd/ftab058)

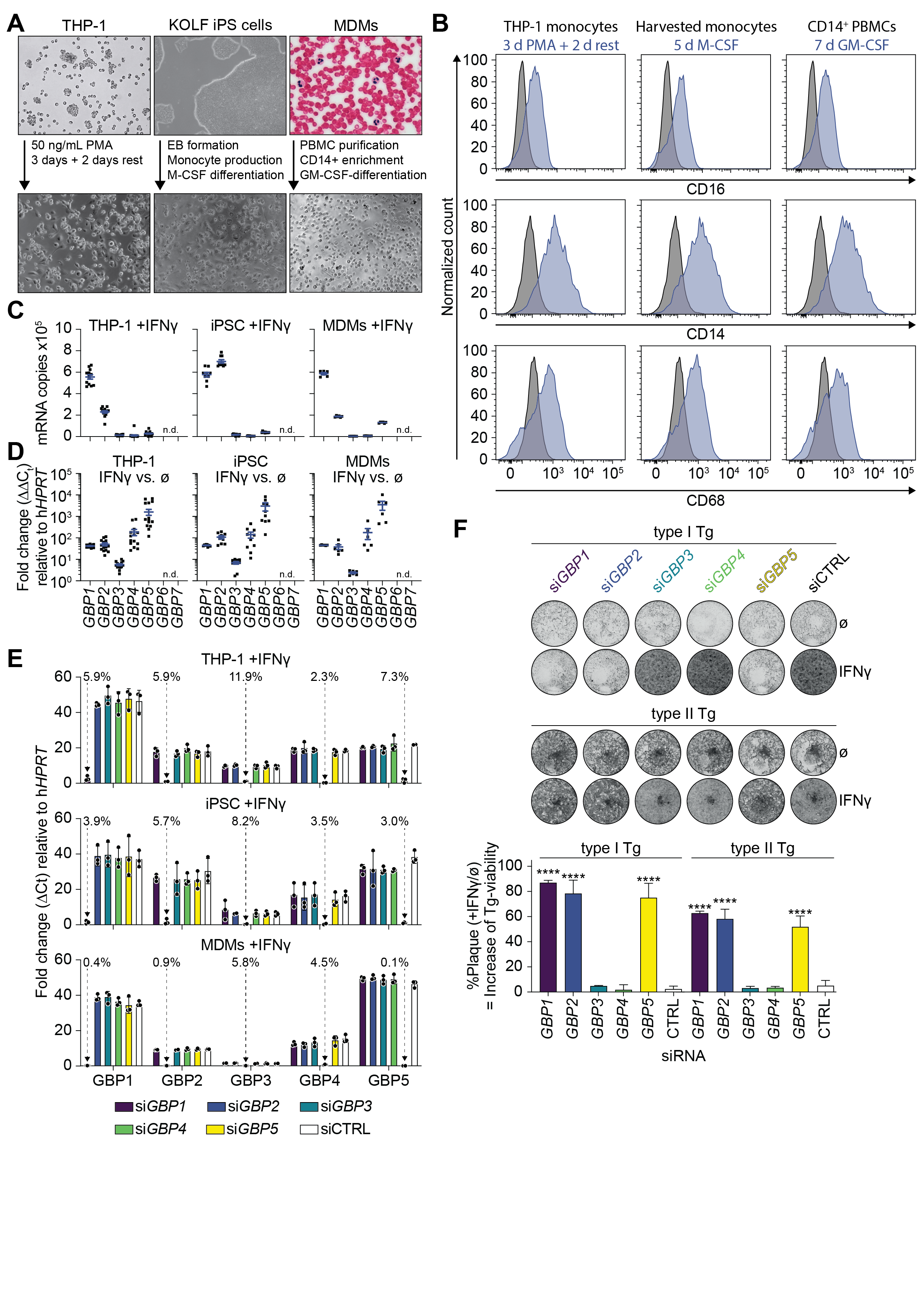

Supplement: ftab058_Supplemental_Files [file ftab058_supplemental_files.zip › Fisch_et_al_P&D_Figure_S1.png]

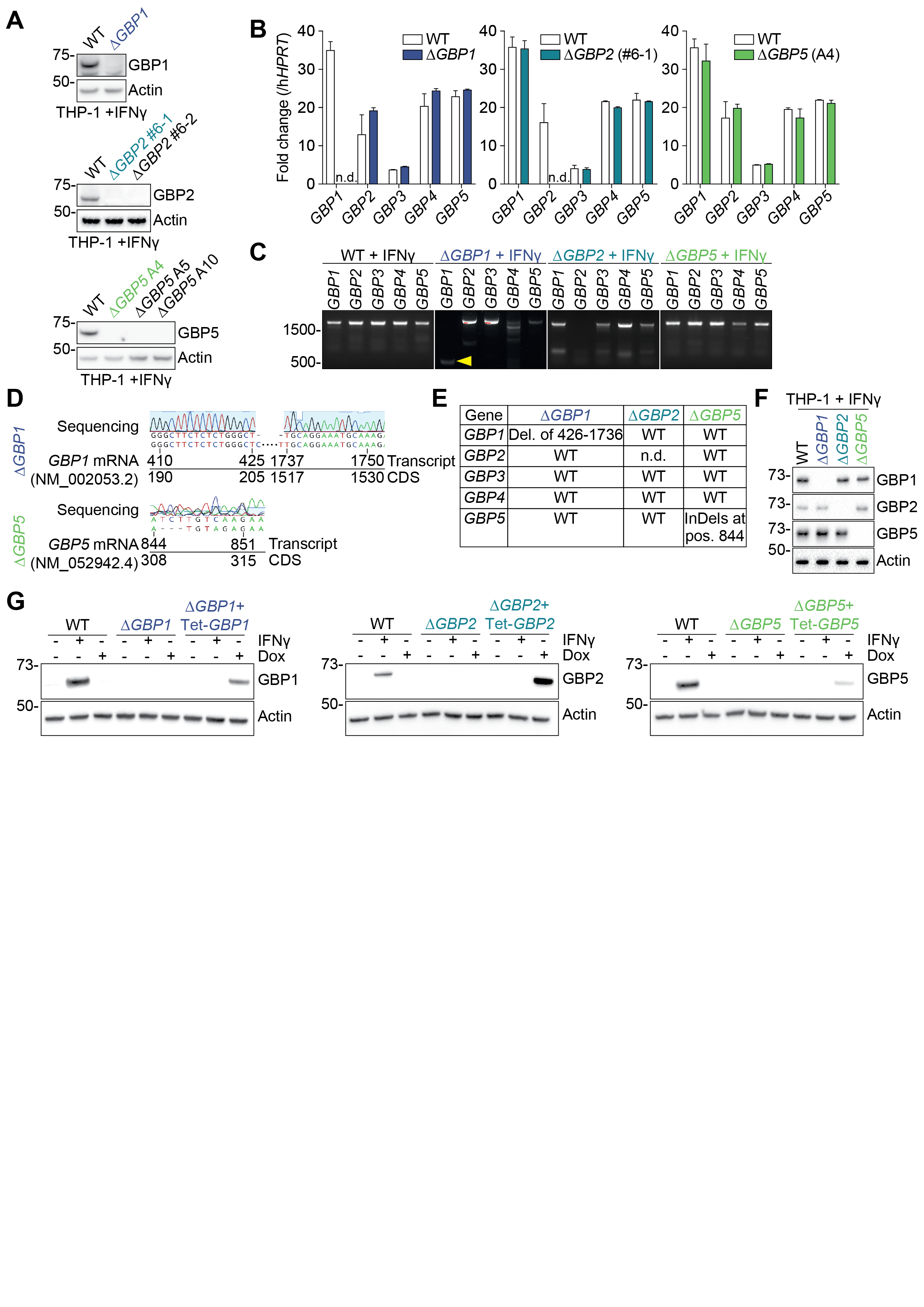

Supplement: ftab058_Supplemental_Files [file ftab058_supplemental_files.zip › Fisch_et_al_P&D_Figure_S2.png]

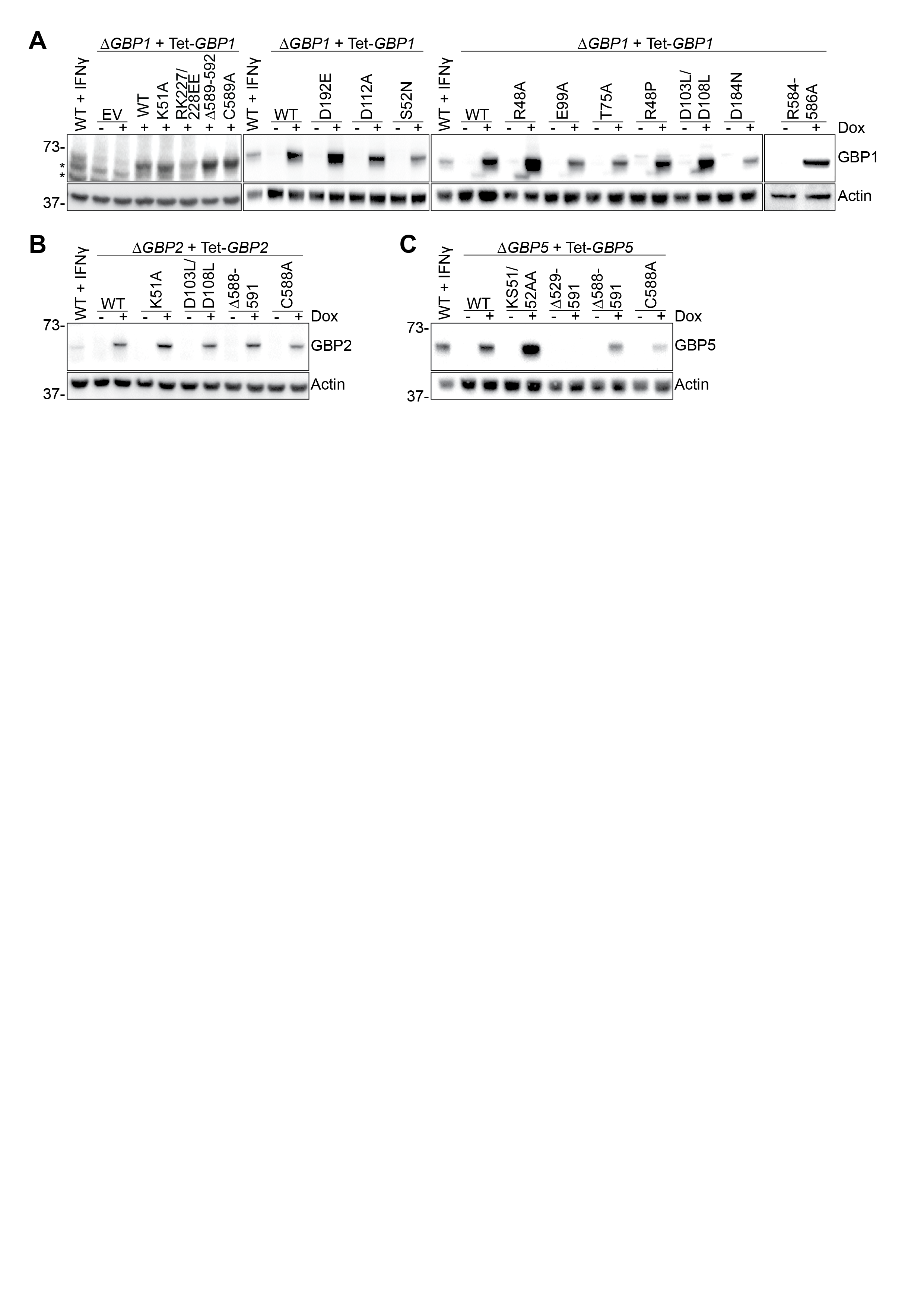

Supplement: ftab058_Supplemental_Files [file ftab058_supplemental_files.zip › Fisch_et_al_P&D_Figure_S3.png]

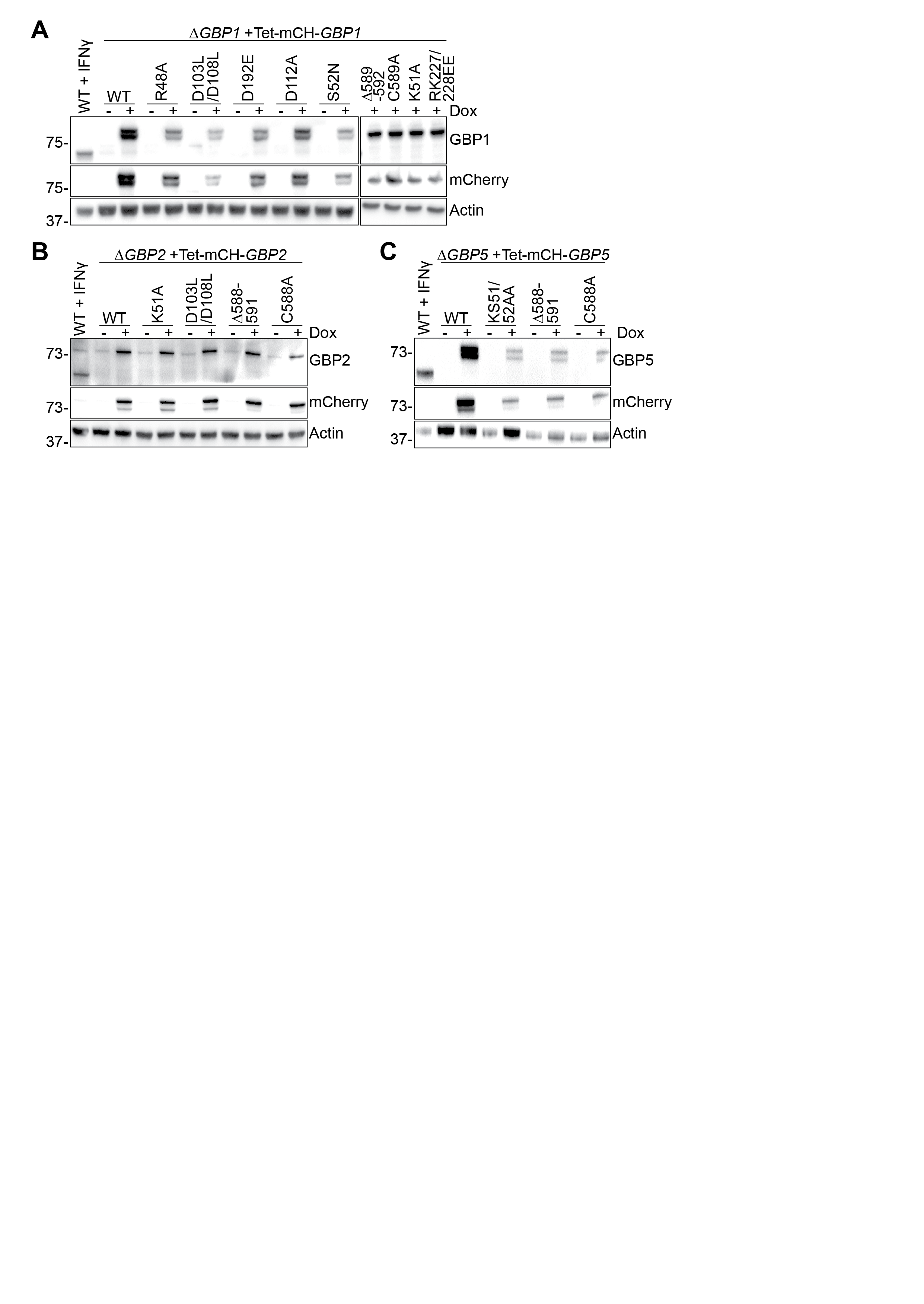

Supplement: ftab058_Supplemental_Files [file ftab058_supplemental_files.zip › Fisch_et_al_P&D_Figure_S4.png]
